# Genomewide m^6^A Mapping Uncovers Dynamic Changes in the m^6^A Epitranscriptome of Cisplatin-Treated Apoptotic HeLa Cells

**DOI:** 10.3390/cells11233905

**Published:** 2022-12-02

**Authors:** Azime Akçaöz Alasar, Özge Tüncel, Ayşe Bengisu Gelmez, Buket Sağlam, İpek Erdoğan Vatansever, Bünyamin Akgül

**Affiliations:** Noncoding RNA Laboratory, Department of Molecular Biology and Genetics, İzmir Institute of Technology, 35430 Urla İzmir, Turkey

**Keywords:** apoptosis, cisplatin, epitranscriptomics, m^6^A, HeLa

## Abstract

Cisplatin (CP), which is a conventional cancer chemotherapeutic drug, induces apoptosis by modulating a diverse array of gene regulatory mechanisms. However, cisplatin-mediated changes in the m^6^A methylome are unknown. We employed an m^6^A miCLIP-seq approach to investigate the effect of m^6^A methylation marks under cisplatin-mediated apoptotic conditions on HeLa cells. Our high-resolution approach revealed numerous m^6^A marks on 972 target mRNAs with an enrichment on 132 apoptotic mRNAs. We tracked the fate of differentially methylated candidate mRNAs under *METTL3* knockdown and cisplatin treatment conditions. Polysome profile analyses revealed perturbations in the translational efficiency of *PMAIP1* and *PHLDA1* transcripts. Congruently, *PMAIP1* amounts were dependent on *METTL3*. Additionally, cisplatin-mediated apoptosis was sensitized by *METTL3* knockdown. These results suggest that apoptotic pathways are modulated by m^6^A methylation events and that the *METTL3–PMAIP1* axis modulates cisplatin-mediated apoptosis in HeLa cells.

## 1. Introduction

Apoptosis is a type of programmed cell death that is required to maintain the delicate balance between survival and cell death as part of cell and tissue homeostasis. Characterized by cytoplasmic shrinkage, chromatin condensation, nuclear fragmentation, and apoptotic bodies, it is highly useful in eliminating damaged or unneeded cells without generating an inflammatory response [1]. Thus, apoptotic processes are targeted by various chemotherapeutic drugs. For example, cisplatin induces apoptosis in cancer cells by inducing DNA lesions through intra- and inter-strand crosslinks in DNA [2,3]. DNA lesions interfere with proper DNA replication and transcription, leading to the activation of DNA-damage response (DDR) and p53. p53 then transcriptionally coordinates the expression of genes that primarily trigger the intrinsic pathway of apoptosis as well as the extrinsic pathway [4]. However, the efficacy of drugs plummets dramatically due to drug resistance, which constitutes a major challenge in clinics for the proper treatment of cancer patients. It is therefore imperative to understand the molecular mechanism of cisplatin-induced apoptosis to develop better strategies against drug resistance.

In an analogy to DNA and proteins, the chemical composition of RNA is modified co- or post-transcriptionally through epitranscriptomic mechanisms that play a vital role in the fate of modified RNAs. Of the existing 170 different types of epitranscriptomic modifications, the *N*^6^-methyladenosine (m^6^A) modification is the most abundant one with the presence of 0.1–0.4% of all adenosines in cellular RNAs [5]. Antibody-based enrichment coupled with high-throughput sequencing has uncovered m^6^A-methylated peaks not only on abundant tRNAs and rRNAs, but also on mRNAs [6,7]. Cell- and tissue-specific m^6^A modifications on mRNAs modulate a diverse array of cellular processes in health and disease. The emerging evidence suggests that proteins and enzymes that orchestrate cellular m^6^A dynamics control apoptosis under various cellular settings. For example, depletion of *METTL3* leads to an increase in the rate of apoptosis by reducing the translation of MYC, BCL2 and PTEN in leukemia cells or by BCL2 translation reduction in breast cancer cells [8,9]. The demethylase FTO, on the other hand, suppresses the rate of apoptosis in human acute myeloid leukemia cells [10]. There are also reports on the modulatory effect of m^6^A reader proteins, such as IGF2BP1, on apoptosis [11]. However, a complete m^6^A methylome of mRNAs under apoptotic conditions has not yet been reported.

We used cisplatin, a universal cancer chemotherapeutic drug, as an inducer of apoptosis to profile m^6^A mRNA methylome in HeLa cells. Our analyses identified differential m^6^A methylation of 972 mRNAs. Interestingly, 132 of m^6^A-methylated mRNAs are associated with apoptosis as revealed by gene ontology analyses. Further analyses showed that cisplatin-induced m^6^A marks do not change the abundance of candidate mRNAs tested. However, we detected changes in the translational efficiencies of differentially methylated candidate mRNAs as revealed by polysome profiling. This observation was further supported by a corresponding increase in the protein amount, suggesting a potential role for the *METTL3–PMAIP1* axis in apoptosis.

## 2. Materials and Methods 

### 2.1. Cell Culture, Drug Treatments and Analysis of Apoptosis

HeLa cells, purchased from DSMZ GmbH (Leibniz, Germany), and an ME-180 (HTB-33) cell line, purchased from ATCC (Manassas, VA, USA), were cultured in RPMI 1640 (Gibco, ThermoFischer, Waltham, MA, USA) and McCoy’s 5A (Lonza, Switzerland) supplemented with 2 mM L-glutamine, 10% fetal bovine serum (FBS) (Gibco, ThermoFischer, Waltham, MA, USA) in a humidified atmosphere of 5% CO_2_ at 37 °C. Cisplatin (Santa Cruz Biotechnology, Dallas, TX, USA) treatments were carried out in triplicates essentially as described previously [12]. 0.1% (*v*/*v*) DMSO was used as a negative control for cisplatin. Treated cells were trypsinized by 1X Trypsin-EDTA (Gibco, ThermoFischer, Waltham, MA, USA) and washed in 1X cold PBS (Gibco, ThermoFischer, Waltham, MA, USA). Cell pellets were stained with Annexin V-PE (BD Biosciences, Franklin Lakes, NJ, USA) and 7AAD (BD Biosciences, Franklin Lakes, NJ, USA) in the presence of 1X Annexin binding buffer (BD Biosciences, Franklin Lakes, NJ, USA) followed by incubation at room temperature for 15 min in the dark. The rates of Annexin V and/or 7AAD-positive cells were quantified by a FACSCanto flow cytometer (BD Biosciences, Franklin Lakes, NJ, USA).

### 2.2. Construction of Overexpression Vector and Cell Transfection

*METTL3* cDNA was amplified by Q5*^®^* High-Fidelity DNA Polymerase (New England Biolabs, Ipswich, MA, USA) with primers harboring a *Nhe*I-*Not*I restriction site (Table 1, METTL3-OE). The 1743-bp PCR product was cloned into pcDNA3.1+ plasmid to obtain pcDNA3.1(+)+METTL3. The sequence of the insert was verified by Sanger sequencing. The pcDNA3.1(+) was used as the negative control. Plasmids were isolated using an endotoxin-free plasmid isolation kit (Macherey-Nagel, Dueren, Germany). A total of 75,000 cells/well HeLa cells were seeded in 6-well plates. After overnight incubation, cells were transfected with 1500 ng DNA/well using the Fugene HD transfection reagent (Promega, Madison, WI, USA) in a 2-mL final volume. Media was changed 1 h post-transfection in overexpression experiments.

0.6 × 10^6^ HeLa cells were seeded into 10 cm dishes (Sarstedt, Nümbrecht, Germany) and incubated overnight prior to transfection with 25 nM non-target pool (si-NC) or METTL3 siRNA (si-METTL3) (Dharmacon, Lafayette, CO, USA). DharmaFECT transfection reagent was mixed with siRNAs in a ratio of 2:1 (*v*/*v*) in an 800-µL serum-free medium and incubated for 5 min at room temperature. The DharmaFECT solution was added into the siRNA tube and incubated for 20 min at room temperature. The mixture was then combined with an 8-mL medium by gently dropping over cells.

### 2.3. Total RNA Isolation and qPCR

Total RNA isolation was performed using TRIzol^TM^ reagent (Invitrogen, ThermoFischer, Waltham, MA, USA) according to the manufacturer’s protocol. Trace DNA contamination was eliminated by treating RNAs with TURBO DNA-free^TM^ kit (ThermoFisher Scientific, Waltham, MA, USA) according to the manufacturer’s instructions.

For qPCR analyses, reverse transcription was conducted with RevertAid first strand cDNA synthesis kit according to the manufacturer’s instructions (ThermoFisher Scientific, Waltham, MA, USA). cDNA was prepared using 2 µg of total RNA and diluted 1:10 in nuclease-free water. qPCR reactions were set up in triplicate with GoTaq^®^ qPCR Master Mix (Promega, Madison, WI, USA) and run in a Rotor-Gene Q machine (Qiagen, Hilden, Germany). The primer sequences are listed in Table 1. All reactions were incubated at 95 °C for 2 min as initial denaturation, 45 cycles of denaturation at 95 °C for 15 s and annealing at 60 °C for 1 min following a melting step. GAPDH was used to normalize the qPCR data.

### 2.4. m^6^A-eCLIP-seq and Bioinformatic Analysis

Three biological replicates of total RNAs isolated from DMSO (control) and cisplatin-treated HeLa cells were subjected to m^6^A-eCLIP-seq by Eclipse Bioinnovations (San Diego, CA, USA) according to the published protocols [13]. The data were deposited to the Genome Expression Omnibus (GEO) with the accession number GSE188580.

Data analysis was performed by Eclipse Bioinnovations using their standard pipeline [14]. Briefly, UMI sequences (the first 10 bases) were demultiplexed from each read by UMI-tools [15]. Quality control of reads and adaptor trimming from 3′ ends was assessed by FastQC: v. 0.11.5 [16] and Cutadapt: v. 1.15 tools [17], respectively. After the removal of contaminating sequences (e.g., rRNA and/or other repetitive sequences) by UMI-tools, the remaining reads were aligned to the human GRCh38/hg38 reference genome utilizing STAR: v. 2.6.0c. Peaks of m6A modification (cluster of reads) were firstly pinpointed by CLIPper tool (https://github.com/YeoLab/clipper, accessed on 1 December 2022) [18,19] and the reproducibility of clusters among biological triplicates of each sample was determined by IDR analysis [20]. The log_2_ fold change was calculated in two steps: (i) log_2_ fold change in IP relative to its corresponding input for each sample, (ii) log2 fold change in cisplatin-treated samples relative to the corresponding DMSO control, and vice versa. Further analysis for determination of crosslink sites at a single nucleotide resolution in IP-enriched samples relative to their inputs was performed by PureCLIP: v. 1.3.1 [14]. The validation of crosslink sites was evaluated through both reproducibility of m^6^A sites among three replicates and determination of the DRACH motif in which m^6^A sites tend to be situated [21]. Finally, each crosslink site was appointed to the gene and feature type based on the GENCODE Release 32 (GRCh38.p13). Further analyses of determined feature types and crosslink sites such as the relative frequency of peaks that map to each feature, Metagene Plot, Metaintron Plot, and Homer Motif analysis, were carried out by Eclipse Bioinnovation.

Differentially methylated RNAs were further interrogated by gene ontology (GO) enrichment analysis, KEGG enrichment analysis, and a Reactome pathway platform (the Gene Ontology Consortium, 2019, [22]).

### 2.5. Western Blotting

Total protein was extracted from cells using 1X RIPA buffer (Cell Signaling Technology, Danvers, MA, USA). Subsequently, 25 µg total protein extract was fractionated on a 10% SDS-PAGE and transferred onto PVDF membranes (ThermoFisher Scientific, Waltham, MA, USA). The membrane was blocked with 5% non-fat dry milk in 0.2% Tween-20 in Tris-buffered saline for 1 h at room temperature. The primary antibodies for *METTL3, METTL14, FTO, RBM15, PMAIP1, Caspase-9, Caspase-3* and *β-actin* (Cell Signaling Technology, Danvers, MA, USA) and secondary antibodies were used in 1:1000 and 1:10,000 dilution, respectively. The chemiluminescent signals were quantitated by the ImageJ program where each signal was normalized to β-actin.

### 2.6. Polysome Analysis

Polysome profiling was performed according to a published procedure [23]. Following centrifugation at 1200 RPM for 10 min, the cells were homogenized in lysis buffer [100 mM NaCl, 10 mM MgCl_2_, 30 mM Tris-HCl (pH 7), 1% Triton X-100, 1% NaDOC, 100 µg/mL cycloheximide (Applichem, Darmstadt, Germany) and 30 U/mL RNase Inhibitor (Promega, Madison, WI, USA)]. The cell pellets were sheared to homogenization by passing the lysate through a 26 G needle at least 15 times and incubated on ice for 8 min. The lysates were then centrifuged at 12,000× g at 4 °C for 8 min. The supernatants were layered over 5–70% (*w*/*v*) sucrose gradients [100 mM NaCl, 10 mM MgCl_2_, 30 mM Tris-HCl (pH 7.0), 200 U RNase inhibitor (Promega, Madison, WI, USA)] prepared by using an ISCO Teledyne (Lincoln, NE, USA) density gradient fractionation system and centrifuged at 27,000 RPM for 2 h 55 min at 4 °C in a Beckman SW28 rotor (Beckman Coulter, Brea, CA, USA). Fractions were collected using the ISCO Teledyne density gradient fractionation system while reading absorbance at A_254_. Fractions were pooled as mRNP, monosomal, light and heavy polysomal sub-groups based on A_254_ readings. The total RNA was phenol-extracted from fractions by using phenol-chloroform-isoamyl alcohol (25:24:1) (Applichem, Darmstadt, Germany).

## 3. Results

### 3.1. Cisplatin Regulates the Expression of the m^6^A Methylation Machinery

**Figure 1:**
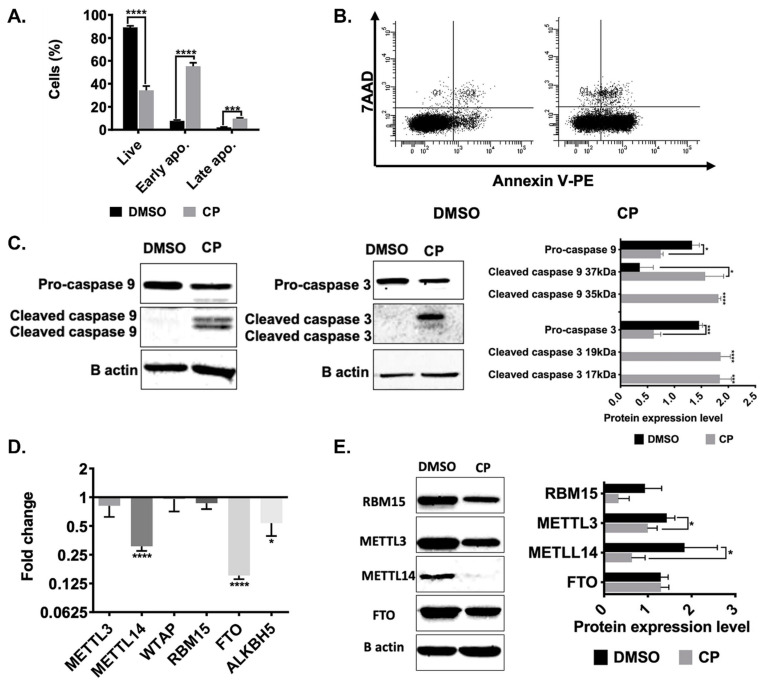
Expression patterns of m^6^A enzymes in cisplatin-treated HeLa cells. 1× 10^6^ HeLa cells were treated with 80 μM cisplatin (CP) and 0.1% (*v*/*v*) DMSO for 16 h. The cells were stained with Annexin V/7AAD and examined by flow cytometry. Population distributions of DMSO- and CP-treated HeLa cells are depicted (**A**) Percentage of live (34%), early (55%) and late apoptosis (9.9%) and (**B**) Dot-blot analysis by flow cytometry after staining with Annexin V-PE and 7AAD. (**C**) Western blot analysis of caspase- 3 and caspase- 9 in HeLa cells treated with 80 µM CP and 0.1% (*v*/*v*) DMSO for 16 h. Equal amounts of total proteins (25 μg/lane) were fractionated through a 10% SDS-PAGE. (**D**) qPCR analysis of gene expression. Results were normalized against GAPDH. (**E**) Western blot analysis. Band intensities were normalized against β-actin, used as a loading control. Experiments were conducted in triplicates. *: *p* ≤ 0.05, ***: *p* ≤ 0.001, ****: *p* ≤ 0.0001 by a two-tailed unpaired t-test.

Cisplatin is a cancer chemotherapeutic drug that is widely used as a universal inducer of apoptosis [24]. We reported previously that cisplatin at a concentration of 80 µM is sufficient to trigger approximately 50% of apoptosis in HeLa cells [25]. Thus, we treated HeLa cells with 80 µM cisplatin for 16 h to attain an early apoptotic rate of 55% (Figure 1A,B). We also analyzed caspase activation for further confirmation of CP-induced apoptosis in HeLa cells. Caspase -3 and caspase -9 were cleaved upon CP treatment as a hallmark of cells undergoing apoptosis (Figure 1C). We then determined the expression levels of major genes involved in m^6^A RNA methylation to assess the impact of cisplatin on the m^6^A methylation machinery. To this extent, we examined the changes in the transcript levels of *METTL3*, *METTL14*, *WTAP*, *RBM15*, *FTO* and *ALKBH5* in cisplatin-treated HeLa cells. Interestingly, cisplatin treatment led to a 3.3- and 6.6-fold reduction in the mRNA levels of *METTL14* and *FTO*, respectively, as determined by qPCR analyses (Figure 1D). We then checked the amount of some of these key proteins. Our western blot analyses showed downregulation of METTL14 in parallel with the qPCR analyses (Figure 1E). Although we did not detect any changes in the mRNA levels of *METTL3* and *RBM15* (Figure 1D), we observed a decrease in the protein levels of RBM15 and METTL3 by 2.8- and 1.4-fold, respectively (Figure 1E).

### 3.2. Cisplatin Modulates Major Changes in the m^6^A RNA Methylome

**Figure 2:**
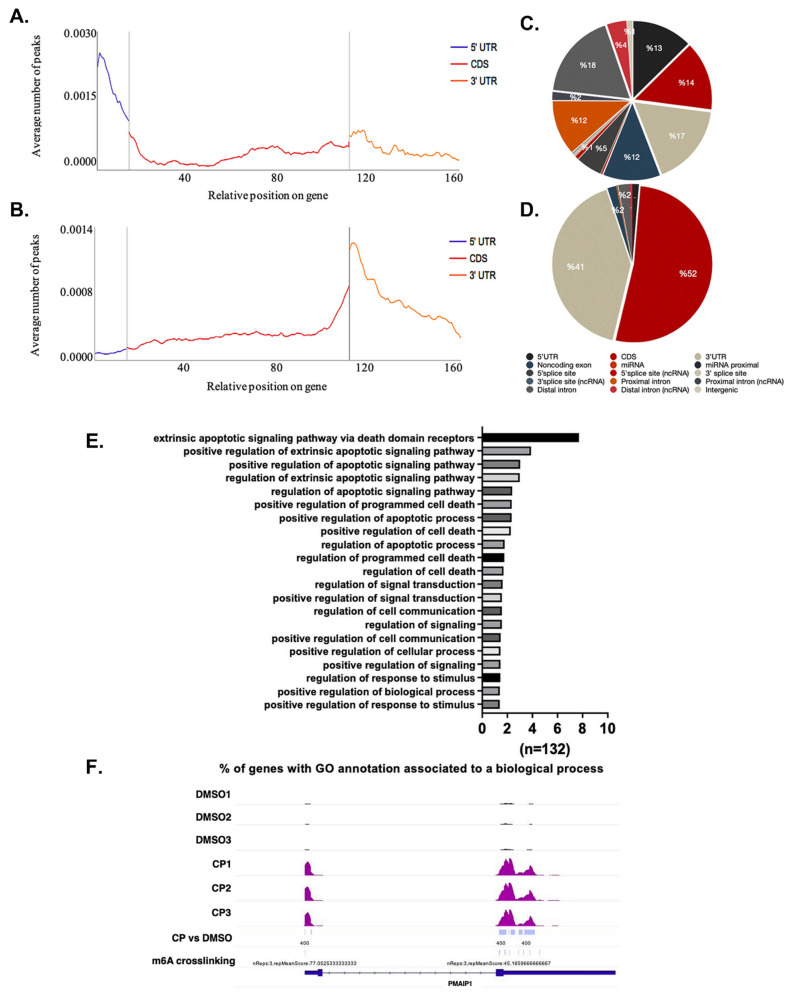
m^6^A RNA methylome profile of cisplatin-treated HeLa cells. The m^6^A methylome of DMSO control and CP-treated HeLa cells were obtained by miCLIP-seq as described in Materials and Methods. Distribution of upregulated (**A**) and downregulated (**B**) m^6^A peaks are shown across all transcripts**.** Pie chart of upregulated (**C**) and downregulated (**D**) m^6^A peaks representing their location on transcripts. Biological replicates: *n * =  3 per group. (**E**) Gene ontology (GO) analysis of differentially m^6^A-methylated transcripts associated with apoptosis. All biological processes were plotted having a false discovery rate (FDR) <  0.05. (**F**) m^6^A methylation profile of *PMAIP1*.

Cisplatin-mediated changes in the mRNA and protein levels of key RNA methylation enzymes suggest substantial perturbations in the cellular RNA m^6^A methylome under apoptotic conditions. Therefore, we employed a genome-wide approach to identify the key RNA m^6^A methylation events in cisplatin-treated HeLa cells. To this extent, we performed an m^6^A miCLIP-seq analysis with three biological replicates of total RNAs isolated from DMSO- and cisplatin-treated HeLa cells. We normalized the changes in RNA m^6^A methylation events against transcript abundance to obtain the differentially m^6^A-methylated transcripts. Our analyses revealed changes in the m^6^A methylation pattern at 7658 adenosine residues with an average number of 12 m^6^A methylation per transcript (Supplementary Table S1). The extent of m^6^A methylation was reduced at 6236 sites while we detected induction in the m^6^A RNA methylation pattern at 1422 sites. We subsequently generated metaintron plots to interrogate the distribution of m^6^A sites throughout transcripts. We noticed an enrichment at the 5′ and 3′ UTRs of transcripts with increased and decreased m^6^A residues, respectively (Figure 2A,B). Additionally, down-regulated m^6^A methylation was predominantly localized to the 3′ UTR and coding sequence of mRNAs, while the up-regulated m^6^A residues have homogenic distribution throughout mRNA (Figure 2C,D). To uncover which transcripts are specifically targeted by the m^6^A methylation machinery under cisplatin-induced apoptotic conditions, we carried out gene ontology analyses with differentially m^6^A-methylated transcripts (Supplementary Table S2). Positive regulation of the apoptotic signaling pathway was listed in number 2 (Supplementary Table S2). We subsequently selected the genes associated with apoptosis (Supplementary Table S3). Strikingly, as many as 132 genes associated with apoptosis were subjected to differential m^6^A methylation (Figure 2E, Supplementary Table S3). We selected differential m^6^A methylation on four candidate mRNAs, namely *PHLDA1, PIDD1, PMAIP1* and *TRAP1*, all of which have been reported to be key players in the modulation of apoptosis [4,26,27,28,29,30]. An IGV screenshot of the methylation profile for *PMAIP1* under DMSO and CP treatment conditions (*n* = 3) is presented in Figure 2F. *PMAIP1* possesses five m^6^A residues under the control DMSO treatment condition. Under cisplatin treatment conditions, we detected only 3 m^6^A marks on *PMAIP1*. Interestingly, cisplatin treatment leads to the addition of an m^6^A mark near the 5′UTR and the removal of three m^6^A residues throughout the transcript. The resulting three m^6^A marks were upregulated 4.23-, 3.5- and 3.67-fold upon cisplatin treatment.

### 3.3. Cisplatin Modulates Translational Efficiency of mRNAs in a METTL3-Dependent Manner

**Figure 3:**
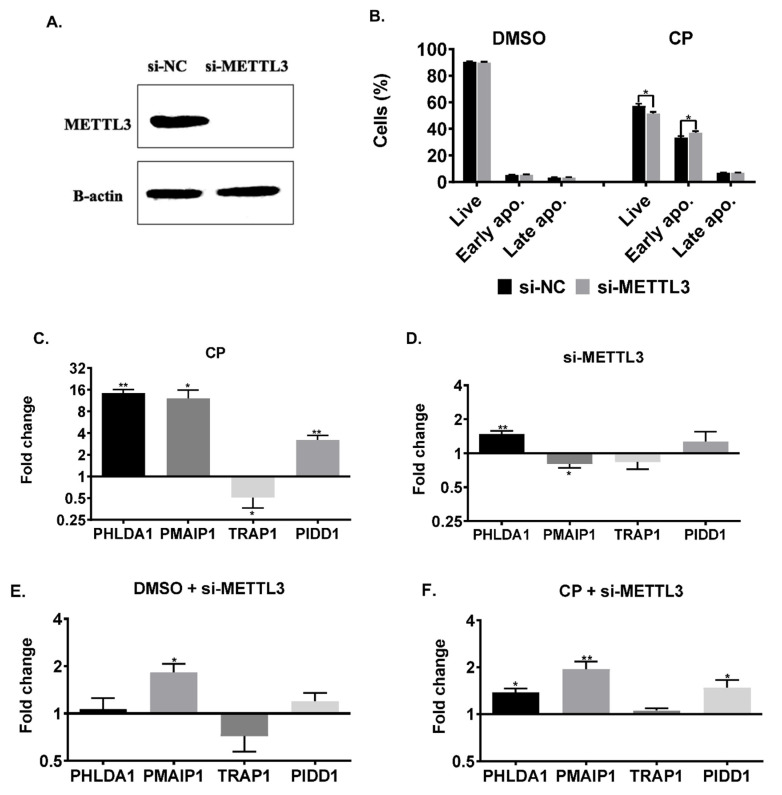
RNA abundance in cisplatin-treated *METTL3* knockdown HeLa cells. (**A**) Western blot showing *METTL3* knockdown in HeLa cells. si-NC was used as negative control. Β-actin was used as loading control. (**B**) Apoptotic population distribution of si-NC- and si-METTL3-transfected cells followed by DMSO (0.05%)- and CP-treatment (40 µM) for 16 h, respectively. qPCR analyses of total RNAs isolated from DMSO- and CP-treated (**C**), si-NC and si-METTL3-transfected cells (**D**), si-METTL3-transfected and DMSO-treated cells (**E**) and si-METTL3-transfected and CP-treated cells (**F**). n = 3 *p* > 0.05, *: *p* ≤ 0.05, **: *p* ≤ 0.01 by two-tailed unpaired *t*-test.

RNA m^6^A methylation may dictate the fate of transcripts both transcriptionally and post-transcriptionally [29]. Therefore, we first examined the impact of RNA m^6^A methylation on transcript abundance in the presence or absence of cisplatin and/or *METTL3*. Although *METTL3* knockdown (Figure 3A) did not change the viability of HeLa cells in the absence of cisplatin (e.g., DMSO treatment), we observed a 3.5% decrease in the percentage of live cells and a 5.8% increase in the percentage of early apoptotic cells (Figure 3B, *p* < 0.05), suggesting that *METTL3* knockdown probably sensitizes HeLa cells to cisplatin-induced apoptosis. Before we investigated the cisplatin inducibility of candidate genes in the absence of *METTL3*, we first interrogated their abundance in cisplatin-treated and *METTL3* knockdown HeLa cells separately. Based on our qPCR analyses, cisplatin treatment led to a 14.3-, 12.1- and 3.2-fold increase in the transcript amount of *PHLDA1, PMAIP1* and *PIDD1*, respectively (Figure 3C). On the other hand, *METTL3* knockdown did not have a major impact on the transcript abundance of any of the candidates tested (Figure 3D). We then measured the mRNA amounts in DMSO- and cisplatin-treated HeLa cells following the knockdown of *METTL3*. Similarly, no major effects were observed on transcript abundance (Figure 3E,F).

**Figure 4:**
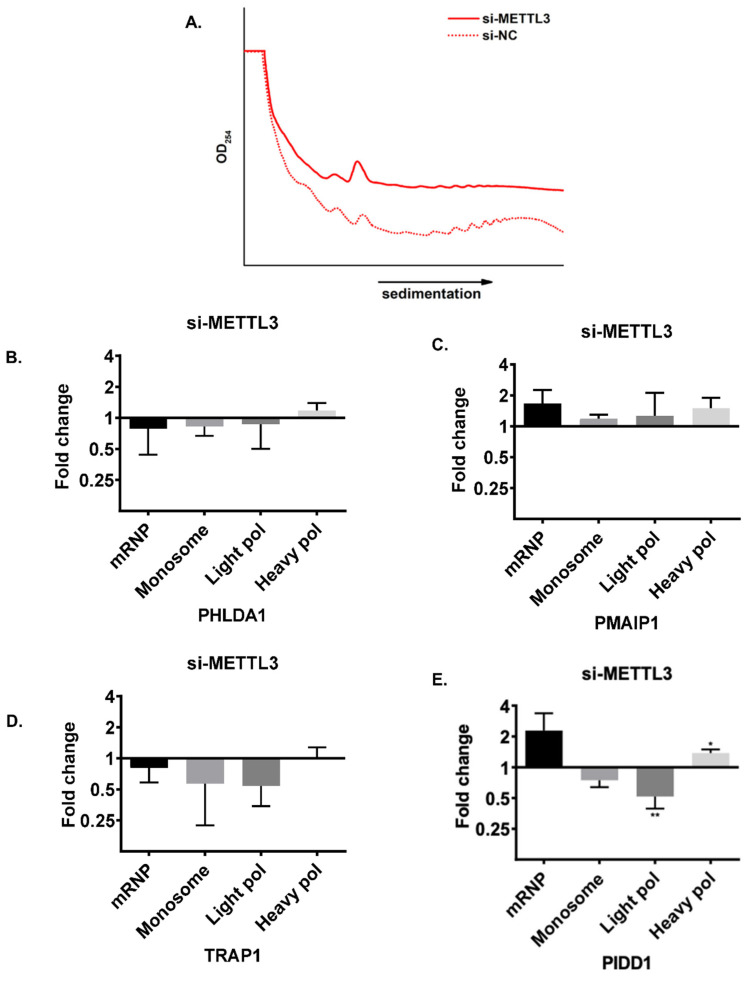
Polysome profiling in *METTL3* knockdown HeLa cells. Polysome profiles of cells transfected with si-METTL3 or negative siRNA (si-NC) (**A**). Total RNAs were phenol-extracted from each fraction collected based on the polysome profile and transcript abundance was measured by qPCR for *PHLDA1* (**B**), *PMAIP1* (**C**), *TRAP1* (**D**) and *PIDD1* (**E**). *n* = 3. *p* > 0.05, *: *p* ≤ 0.05, **: *p* ≤ 0.01 by two-tailed unpaired t-test.

**Figure 5:**
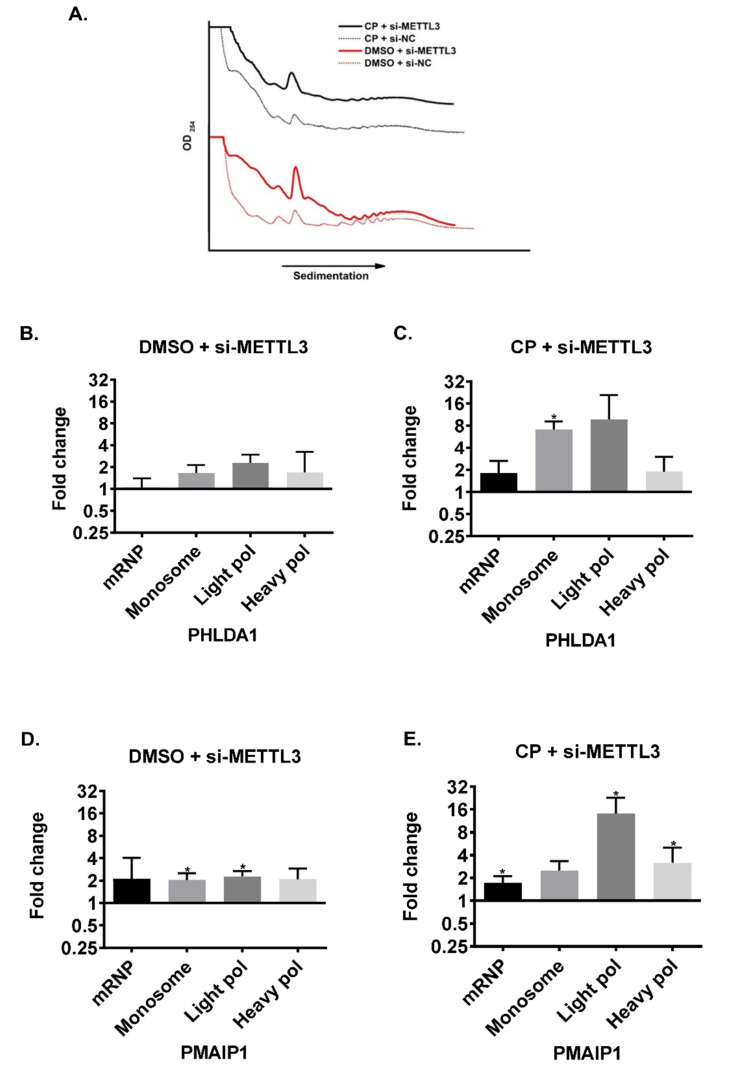

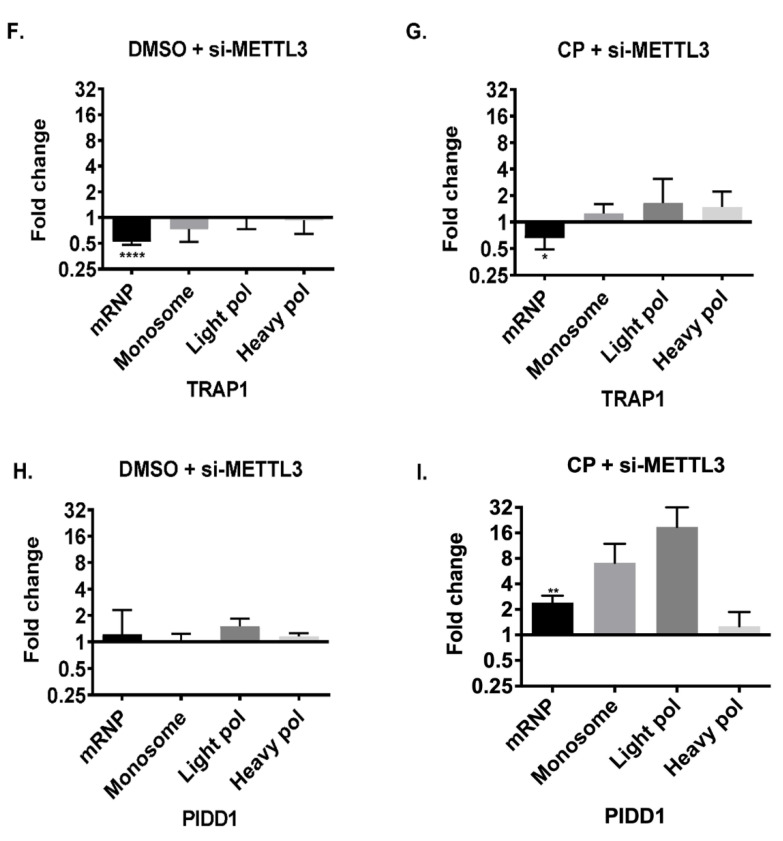
Polysome profiling in cisplatin-treated *METTL3* knockdown HeLa cells. Polysome profiles of cells transfected with si-METTL3 or negative siRNA (si-NC) and treated with 40 µM CP for 16 h (**A**). Total RNAs were phenol-extracted from each fraction and transcript abundance was examined by qPCR for *PHLDA1* (**B**,**C**), *PMAIP1* (**D**,**E**), *TRAP1* (**F**,**G**) and *PIDD1* (**H**,**I**) *n* = 3. *: *p* ≤ 0.05, **: *p* ≤ 0.01, ****: *p* ≤ 0.0001 by two-tailed unpaired *t*-test.

**Figure 6:**
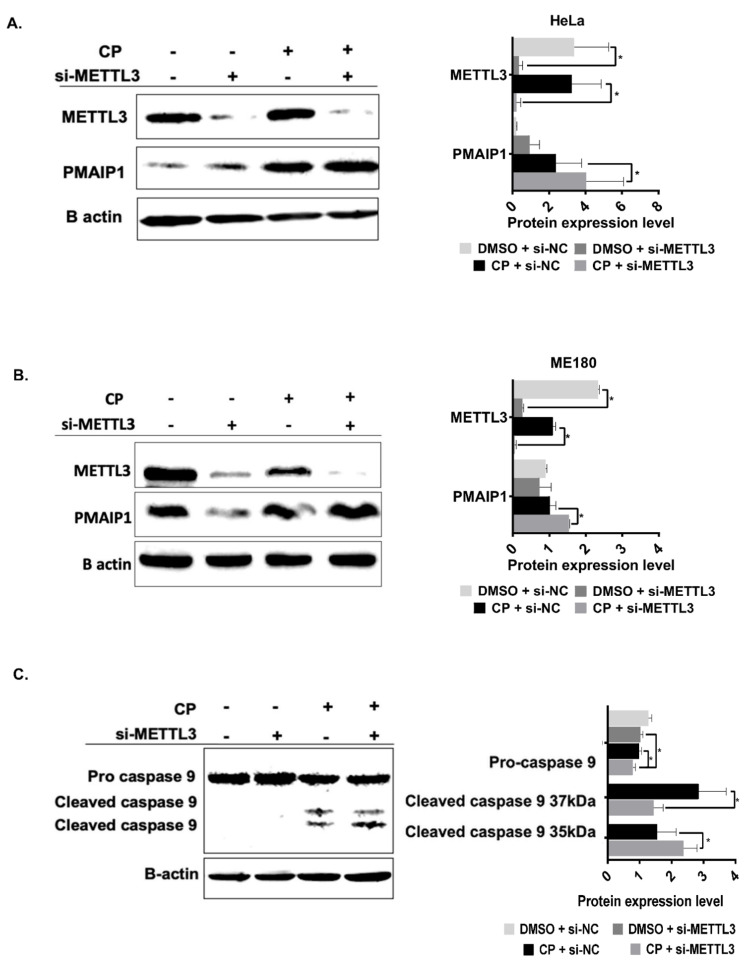

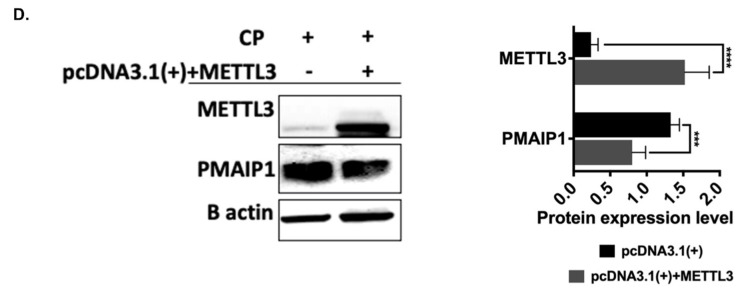
The *METTL3-PMAIP1* axis in *METTL3* knockdown and overexpressed HeLa cells. Constitutive *PMAIP1* protein expression in HeLa (**A**,**C**) and ME-180 (**B**) cells transfected with si-METTL3 and treated with CP as in Figure 3. Overexpression of *METTL3* causes reduction in CP-induced *PMAIP1* (**D**). β-actin was used as loading control. *n* = 3 *: *p* ≤ 0.05 ***: *p* ≤ 0.001, ****: *p* ≤ 0.0001 by two-tailed unpaired *t*-test.

As the transcript abundance of candidate genes does not appear to be influenced by *METTL3* knockdown in cisplatin-treated HeLa cells, we hypothesized that RNA m^6^A methylation could be critical for translational efficiency of target RNAs under cisplatin-induced apoptotic conditions. Thus, we examined the polysome profiles of cells under various conditions as the association of mRNAs with polysomes is a good indicator of their translational efficiency [30]. The knockdown of *METTL3* did not appear to have a discernible effect on the overall polysome profile (Figure 4A; Supplementary Figure S1), excluding any global effect on translation. We then investigated the association of individual mRNAs with actively translating polysomes. To this extent, we first fractionated the cytoplasmic extracts of HeLa cells into four major fractions (1) mRNP fraction composed primarily of non-translated ribonucleoproteins; (2) monosome constituting mRNAs associated with a single ribosome; (3) light polysome that contains mRNAs associated with up to 3 ribosomes; and (4) heavy polysome that contains mRNAs with more than 3 ribosomes. We performed qPCR analyses with phenol-extracted total RNAs from each fraction. Our results exhibited almost no changes in the translational efficiency of *PHLDA1*, *PIDD1*, *PMAIP1* and *TRAP1* mRNAs upon *METTL3* knockdown (Figure 4B–E). We then examined the translational efficiency of these transcripts in cisplatin-treated HeLa cells upon the *METTL3* knockdown to interrogate the potential contribution of *METTL3* to the translational efficiency of transcripts under cisplatin-induced apoptosis. In agreement with our data in Figure 4, the treatment of HeLa cells with DMSO as a control group did not cause any changes in the sedimentation of the transcripts on sucrose gradients upon the *METTL3* knockdown (Figure 5A,B,D,F,H; Supplementary Figure S2). Interestingly, we detected CP-mediated changes in the translational efficiency of *PHLDA1* (Figure 5C, monosome, 7.1-fold, *p* < 0.05), *PMAIP1* (Figure 5E, light polysome, 14.2-fold, *p* < 0.05) and *PIDD1* (Figure 5I, mRNP, 2.4-fold, *p* < 0.05) mRNAs, but not TRAP1 (Figure 5G). A CP-mediated increase in the translational efficiency of *PMAIP1* has caught our attention as *PMAIP1* has been reported to mediate apoptosis by inducing the intrinsic apoptotic pathway [31]. To substantiate our observation with the polysome analysis, we measured the protein amount of *PMAIP1* in CP-treated HeLa cells upon the *METTL3* knockdown. *PMAIP1* was undetectable in DMSO-treated cells. However, CP caused a sharp increase in the protein level (Figure 6A). We then examined the protein level of *PMAIP1* in *METTL3* knockdown cells upon CP treatment. Interestingly, the *METTL3* knockdown resulted in an increase in the protein level of *PMAIP1* compared to the cells transfected with the control siRNA (Figure 6A,B, 1.5-fold, *p* < 0.001). The CP-mediated increase in the protein level of *PMAIP1* in *METTL3-*knockdown cells was accompanied by a parallel increase in the amount of cleaved caspase 9 (Figure 6C). To examine whether the change in the *PMAIP1* amount is specifically due to the knockdown of *METTL3* upon CP treatment, we performed CP treatment in HeLa cells in which *METTL3* was overexpressed. *METTL3* overexpression was able to partially rescue the change in the *PMAIP1* expression as *METTL3* overexpression resulted in a decrease in *PMAIP1* protein levels (Figure 6D).

## 4. Discussion

We report for the first time that the m^6^A methylation machinery plays a fundamental role in modulating cisplatin-mediated apoptosis in HeLa cells (Figure 1). As a drug with pleiotropic effects, cisplatin induces dynamic changes in the m^6^A methylome associated not only with apoptosis but also with stress, growth, migration, etc. (Figure 2). Our genome-wide approach uncovers the fact that the *METTL3-PMAIP1* axis appears to modulate apoptosis through the *METTL3-*mediated changes in *PMAIP1* protein amounts (Figure 5 and Figure 6).

*METTL3* has been reported to play a role in cell death as its knockdown induces apoptosis in HepG2 cells by modulating the *P53* signaling and splicing of isoforms of *MDM2*, *FAS* and *BAX* [6]. *METTL3* was also shown to be involved in the selective recruitment of DNA polymerase to damaged DNA sites in order to orchestrate repair [32]. Additionally, there are reports on the role of ALKBH5-mediated methylation on cisplatin resistance [33,34]. Although we did not detect any change in the rate of apoptosis in *METTL3* knockdown cells, the knockdown sensitized the cells to cisplatin-induced apoptosis (Figure 3B). Our gene expression analyses in apoptotic HeLa cells also revealed major perturbations in the amounts of *METTL3, METTL14* and *RBM15* without any change in *FTO* (Figure 1). Although these observations clearly suggest a critical role for m^6^A methylation in coordinating different types of cell death [35], a complete m^6^A methylome profiling would be needed to gain insight into the extent of dynamic changes in the m^6^A RNA methylome under cisplatin-induced apoptotic conditions. We detected as many as 972 differentially m^6^A-methylated mRNAs, of which 132 were associated with apoptosis (Figure 2). Condition-specific enrichment of m^6^A on mRNAs has been reported previously. For example, m^6^A residues in 5′ UTRs have been associated with a cap-independent translation [36]. On the other hand, m^6^A residues on coding regions (CDs) were reported to induce translation by helping resolve secondary structures [37]. We did not detect any enrichment on any specific regions of mRNAs except for a slight enrichment on the terminal part of 5′ and 3′ UTRs (Figure 2A). 

m^6^A residues determine the fate of mRNAs at both transcriptional and posttranscriptional levels [29]. We first examined the abundance of our candidate mRNAs to probe into the impact of differential m^6^A methylation on the transcription rate and/or mRNA stability. Our qPCR analyses revealed no changes in the mRNA abundance of *PHLDA1, PMAIP1, TRAP1* and *PIDD1* (Figure 3). However, we observed a strong association between *METTL3* and translational efficiency of these mRNAs especially under cisplatin treatment conditions (Figure 5). Although *METTL3* is reported to promote translation in human cancer cells independent of its catalytic activity and m^6^A readers [38], the *METTL3* knockdown resulted in a better association of our candidate mRNAs with polysomes, especially *PHLDA1*, *PIDD1* and *PMAIP1*, under cisplatin treatment conditions (Figure 5). It is interesting that the *METTL3* knockdown did not influence the extent of polysome association of these RNAs under control DMSO treatment (Figure 4 and Figure 5).

**Figure 7:**
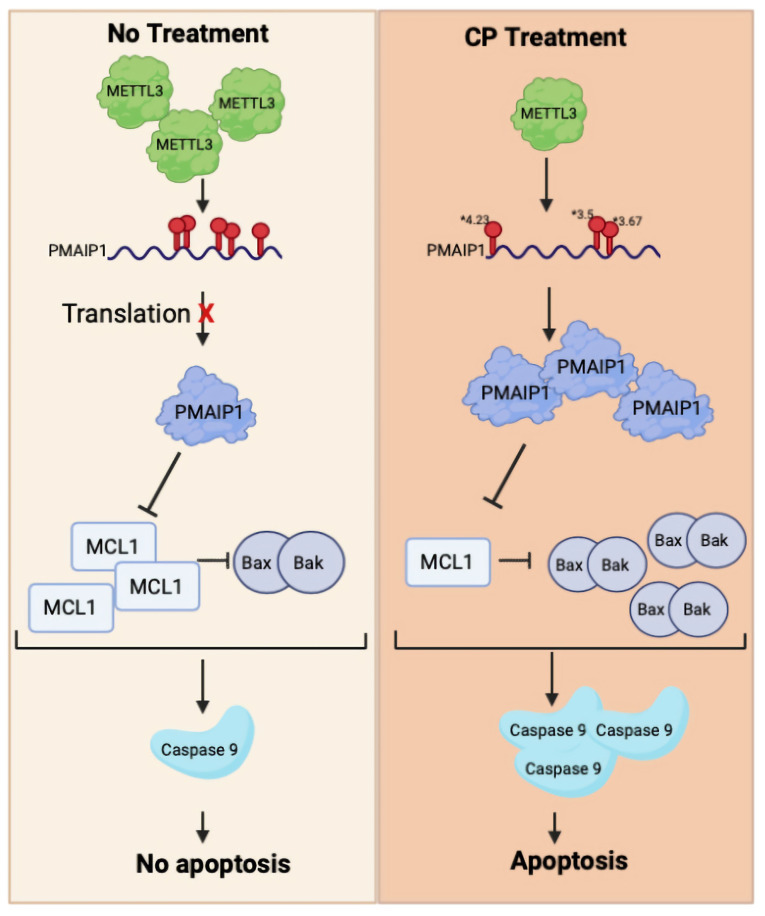
Working model. Under control conditions, *PMAIP1* mRNA possesses m^6^A marks at five different sites. Cisplatin treatment downregulates *METTL3* and mediates demethylation of three m^6^A residues. However, 5′UTR of *PMAIP1* is methylated and *PMAIP1* translation is enhanced. Translationally enhanced *PMAIP1* may then promote apoptosis by inhibiting MCL1 [41], suggesting a novel *METTL3–PMAIP1* axis that may modulate apoptosis under cisplatin treatment conditions. * Fold of induction.

*PMAIP1* is a proapoptotic protein that targets MCL1 or BCL2A1 proteins for degradation [39]. As a p53-responsive gene, *PMAIP1* induces apoptosis in HeLa cells by activating caspase-9 [31]. Thus, we examined whether the enhanced translational efficiency of *PMAIP1* in *METTL3* knockdown cells results in an increase in its protein amount. Expectedly, the *METTL3* knockdown leads to an increase in the *PMAIP1* amount both in HeLa and ME-180 cells (Figure 6A,B). Additionally, we detected an elevation in the amount of cleaved caspase-9 in cisplatin-treated HeLa cells upon the *METTL3* knockdown. It is extremely interesting that the *PMAIP1* is targeted by the m^6^A machinery both transcriptionally [40] and post-transcriptionally (Figure 6). Additionally, miCLIP results showed that the *PMAIP1* has a m^6^A residue near 5′UTR in cisplatin-treated HeLa cells (Figure 7). It is interesting that cisplatin treatment leads to the addition of a m^6^A mark on one site while removing m^6^A marks on other sites. In a stress-induced translational response, mRNA containing an m^6^A modification in their 5′UTR leads to a translation via the eukaryotic initiation factor 3 (eIF3) in a cap-independent manner [36]. A high translation efficiency of the *PMAIP1* may be explained by an m^6^A residue within the 5′UTR under cisplatin treatment. Future experiments combining a depletion in m^6^A residue within the 5′UTR of *PMAIP1* and its interaction with eIF3 will provide more insight into the *PMAIP1* level in regulating the m^6^A and translation.

## Figures and Tables

**Table 1:** The list of primers used in qPCR analyses. OE, overexpression primers.

Genes	Forward 5′-3′	Reverse 5′-3′
METTL3	AGATGGGGTAGAAAGCCTCCT	TGGTCAGCATAGGTTACAAGAGT
METTL14	GAGTGTGTTTACGAAAATGGGGT	CCGTCTGTGCTACGCTTCA
WTAP	TTGTAATGCGACTAGCAACCAA	GCTGGGTCTACCATTGTTGATCT
RBM15	AAGATGGCGGCGTGCGGTTCCGCTGTG	AAGTTCACAAAGGCTACCCGCTCATCC
FTO	CTTCACCAAGGAGACTGCTATTTC	CAAGGTTCCTGTTGAGCACTCTG
ALKBH5	TCCAGTTCAAGCCTATTCG	CATCTAATCTTGTCTTCCTGAG
YTHDF1	TAAGGAAATCCAATGGACGG	TTTGAGCCCTACCTTACTGGA
YTHDF2	CCTTAGGTGGAGCCATGATTG	TCTGTGCTACCCAACTTCAGT
YTHDF3	TGACAACAAACCGGTTACCA	TGTTTCTATTTCTCTCCCTACGC
YTHDC1	TCAGGAGTTCGCCGAGATGTGT	AGGATGGTGTGGAGGTTGTTCC
YTHDC2	GTGTCTGGACCCCATCCTTA	CCCATCACTTCGTGCTTTTT
IGF2BP1	TAGTACCAAGAGACCAGACCC	GATTTCTGCCCGTTGTTGTC
IGF2BP2	ATCGTCAGAATTATCGGGCA	GCGTTTGGTCTCATTCTGTC
IGF2BP3	AGACACCTGATGAGAATGACC	GTTTCCTGAGCCTTTACTTCC
PRRC2A	AGGGCAAGTCCTTAGAGATCC	TTCAGGCTTGGAAGGTTGGC
FMR1	CAGGGCTGAAGAGAAGATGG	ACAGGAGGTGGGAATCTGA
HNRNPA2B1	AGCTTTGAAACCACAGAAGAA	TTGATCTTTTGCTTGCAGGA
HNRNPC	TAAGGAAATCCAATGGACGG	TTTGAGCCCTACCTTACTGGA
HNRNPG	TAAGGAAATCCAATGGACGG	TTTGAGCCCTACCTTACTGGA
PHLDA1	CTTCACTGTGGTGATGGCAGAG	CCTGACGATTCTTGTACTGCACC
PMAIP1	CTCTGTAGCTGAGTGGGCG	CGGAAGTTCAGTTTGTCTCCA
PIDD1	TGTTCGAGGGCGAAGAGTTC	TCCAGAGTGGTGGTCACGTA
TRAP1	CAGGGTTCCACTTCCAAACA	TGGAGATCAGCTCCCGTATAA
METTL3-OE	CTAGCTAGCATGTCGGACACGTGGA	CTAGCGGCCGCCTATAAATTCTTAGG
GAPDH	ACTCCTCCACCTTTGACGC	GCTGTAGCCAAATTCGTTGTC

## Data Availability

The data were deposited to the Genome Expression Omnibus (GEO) with the accession number GSE188580.

## Supplementary Materials

The following supporting information can be downloaded at: https://www.mdpi.com/article/10.3390/cells11233905/s1. Figure S1. Representative profile for area calculation (A). The numbers represent the calculated area of mRNP, monosome and polysomal volumes. Each fraction was calculated, and statistically analyzed on Graphpad (B). One of the replicates has been presented in the main manuscript. Two other replicates for si-METTL3 and si-NC pairs were illustrated in C and D. Figure S2. The calculated area of the profiles of DMSO and CP treated si-NC and si-METTL3 cells were shown as bar graphs (A). One of the replicates has been presented in the main Manuscript. The translational profiles of two other replicates were represented in B and C. Table S1: DOWNREGULATED m6A. Table S2: GEO analysis. Table S3: Differentially methylated apoptotic genes.
