# Supplementary material for: Genomewide m6A Mapping Uncovers Dynamic Changes in the m6A Epitranscriptome of Cisplatin-Treated Apoptotic HeLa Cells
Source: Cells. 2022 Dec 2;11(23):3905. doi: 10.3390/cells11233905 (PMC9738315; doi:10.3390/cells11233905)
Supplement: Supplementary file 1 [file cells-11-03905-s001.zip › cells-1856677-supplementary Figures S1-S2.pdf]

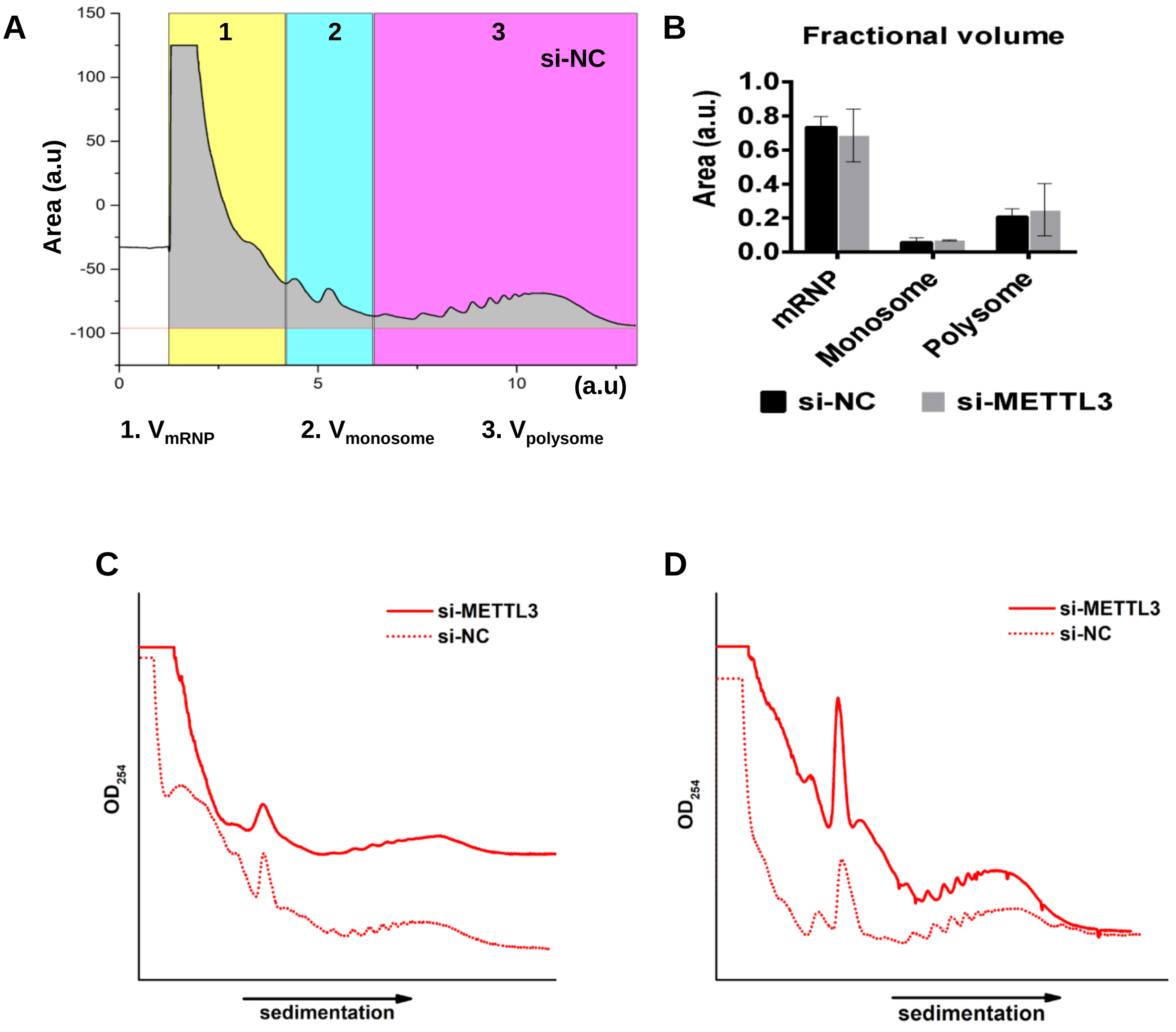

Figure S1. Representative profile for area calculation (A). The numbers represent the calculated area of mRNP, monosome and polysomal volumes. Each fraction was calculated, and statistically analyzed on Graphpad (B). One of the replicates has been presented in the main manuscript. Two other replicates for si-METTL3 and si-NC pairs were illustrated in C and D.

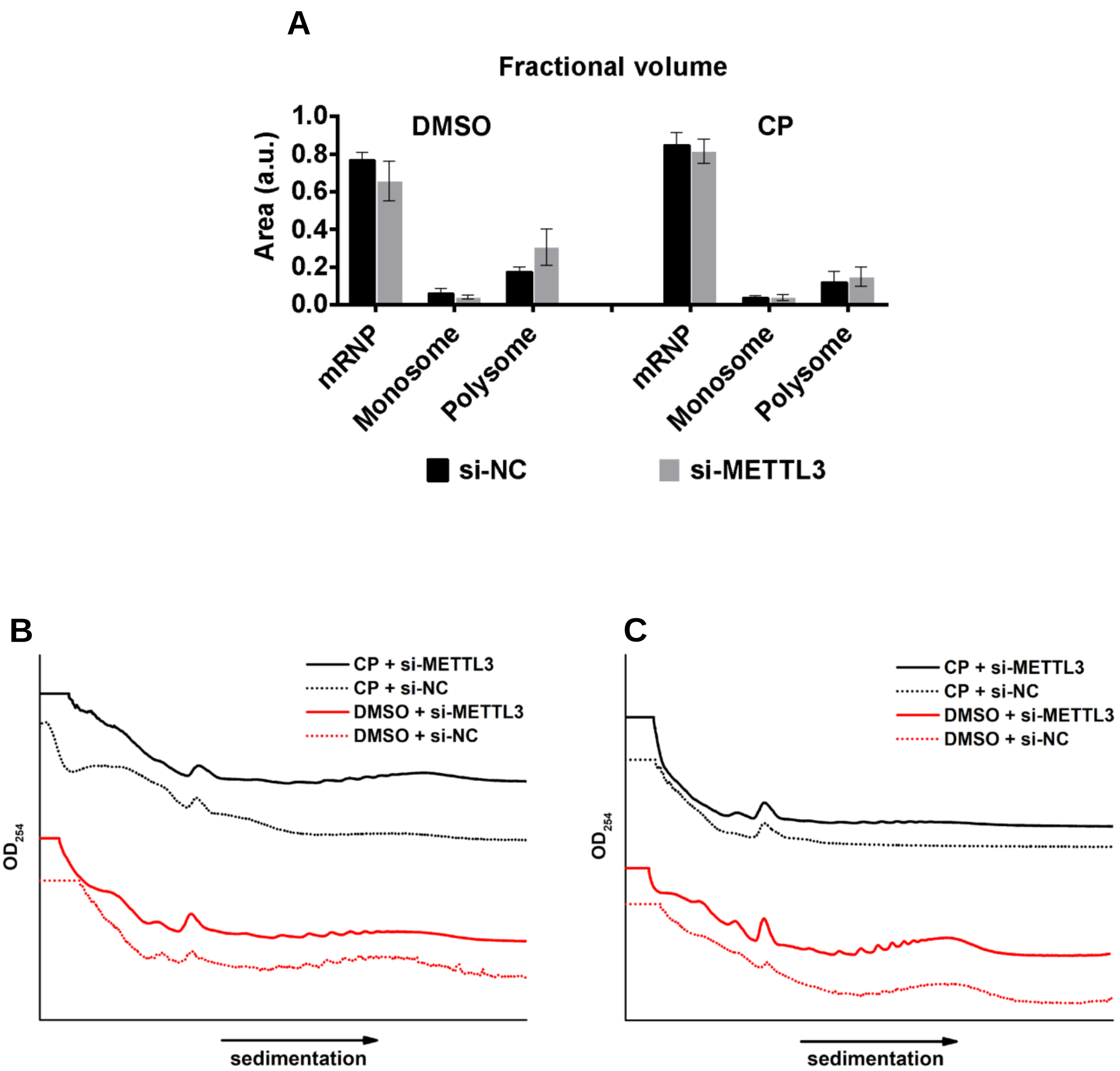

Figure S2. The calculated area of the profiles of DMSO and CP treated si-NC and si-METTL3 cells were shown as bar graphs (A). One of the replicates has been presented in the main Manuscript. The translational profiles of two other replicates were represented in B and C.
